# Correction: Paired competing neurons improving STDP supervised local learning in spiking neural networks

**DOI:** 10.3389/fnins.2025.1641314

**Published:** 2025-06-25

**Authors:** Gaspard Goupy, Pierre Tirilly, Ioan Marius Bilasco

**Affiliations:** Univ. Lille, CNRS, Centrale Lille, UMR 9189 CRIStAL, Lille, France

**Keywords:** Spiking Neural Networks, image recognition, supervised STDP, Winner-Takes-All, intra-class competitive learning

The title of this article was erroneously given as: Paired competing neurons improving STDP supervised local learning in Spiking Neural Networks. The correct title of the article is: Paired competing neurons improving STDP supervised local learning in spiking neural networks.

In the published article, there was a spelling error. A correction has been made to Section 6, Paragraph 2. This sentence previously stated: This includes exploring both feedback connetions (Zhao et al., 2020) and local losses (Mirsadeghi et al., 2021). The corrected sentence appears below: This includes exploring both feedback connections (Zhao et al., 2020) and local losses (Mirsadeghi et al., 2021).

In the published article, there was an error. The argmin function is missing from Equation 6, in the HTML version. The correction has been made to Section 4.1, Paragraph 1, Equation 6.

The original article has been updated.

