# Supplementary material for: Paired competing neurons improving STDP supervised local learning in spiking neural networks
Source: Front Neurosci. 2024 Jul 24;18:1401690. doi: 10.3389/fnins.2024.1401690 (PMC11307446; doi:10.3389/fnins.2024.1401690)
Supplement: Supplementary file 1 [file Data_Sheet_1.PDF]

Supplementary Material

1 TRAINING OF THE FEATURE EXTRACTION NETWORK

To improve image representation before classification, we use a CSNN trained with unsupervised STDP as a feature extraction network. The trainable component of our CSNN is the spiking convolutional layer. Similar to traditional convolutional neural networks, the neurons in the spiking convolutional layer are arranged in two-dimensional maps. Within each map, neurons share weights and thresholds. To avoid the non-local communication required by shared weights, we employ a specific training procedure, with only one active neuron per map (Falez et al., 2019). For each sample, we first extract a random patch from the input image, matching the dimensions of the kernel. Then, we propagate the encoded patch to the active neurons of the convolutional layer. Due to the WTA competition, only the first neuron to fire is updated with the multiplicative STDP (described in the main paper). Then, the thresholds of the active neurons are updated through two adaptation rules. The first one is applied to control the timestamp at which they should fire, which impacts the learnable pattern granularity:

$$V_{\text{th}_i} = \max\left\{\text{Th}_{\text{min}}, V_{\text{th}_i} - \eta_{\text{th}}\left(t_i - t_{\text{target}}\right)\right\},$$

where V_{th_i} represents the threshold of neuron n_i, Th_{min} is the minimum threshold, η_{th} is the learning rate, t_i and t_{target} are the actual and target firing timestamp of the neuron. The second one is applied to ensure homeostasis (i.e. fair competition among neurons) in the layer:

$$\Delta V_{\text{th}_i} = \begin{cases} \eta_{\text{th}} & \text{if } t_i = \min\left\{t_1, \cdots, t_N\right\} \\ \frac{-\eta_{\text{th}}}{N} & \text{o.w.} \end{cases}$$
$$V_{\text{th}_i} = \max\left\{\text{Th}_{\text{min}}, V_{\text{th}_i} + \Delta V_{\text{th}_i}\right\},$$

where N is the number of neurons in competition (i.e. the number of maps). Once the layer is trained, the parameters (weights and thresholds) of the active neurons are fixed and are copied onto the other neurons of the maps.

2 HYPERPARAMETERS

To preprocess MNIST and Fashion-MNIST datasets, we use on-center/off-center coding filters of size 7×7 and standard deviations of 1 and 2 pixels. To preprocess the CIFAR-10 dataset, we use a patch-based whitening method (filters of size 9×9, stride of 2, trained on 10^6 samples). Hyperparameters of the CSNN are described in Table S1. Note that the number of epochs is proportional to the number of filters. Hyperparameters of the classification layer are described in Table S3. Gridsearch-optimized values are presented in Table S4 for SSTDP without weight normalization, in Table S4 for SSTDP with weight normalization, in Table S6 for S2-STDP without weight normalization, in Table S7 for S2-STDP with weight normalization, and in Table S8 for S2-STDP+PCN with weight normalization. In both feature extraction and classification layers, weights are initialized following a normal distribution $N(0.5, 0.01)$ and are clipped in $[0, 1]$ after each update. For more details about the hyperparameters, such as the R-STDP hyperparameters or the gridsearch ranges, please refer to the JSON files located in the `scripts/config/` folder of our source code.

Table S1. Hyperparameters of the CSNN trained with STDP.

Hyperparameter	Value
epochs	25 / 50 / 100
number of filters	16 / 64 / 128
filter size	5×5
stride	1
padding	0
objective time (t_{target})	See Table S2
firing threshold (V_{th})	See Table S2
minimum threshold (Th_{min})	2
threshold learning rate (η_{th})	1
STDP saturation factor (β)	1
STDP learning rates (A^+, A^-)	0.1, -0.1
annealing	0.95
max-pool filter size	4×4
max-pool stride	1
max-pool padding	0

Table S2. Task-dependent hyperparameters of the CSNN trained with STDP.

Hyperparameter	MNIST	Fashion-MNIST	CIFAR-10
objective time (t_{target})	0.75	0.80	0.95
firing threshold (V_{th})	5	5	10

Table S3. Hyperparameters of the classification layer trained with SSTDP-based rules.

Hyperparameter	Value
epochs	100
early stopping	10
annealing	0.98
STDP saturation factor (β)	1
firing threshold (V_{th})	See Tables S4, S5, S6, S7, S8
time gap (g)	See Tables S4, S5, S6, S7, S8
STDP learning rates (A^+, A^-)	See Tables S4, S5, S6, S7, S8
normalization factor (w_{norm})	See Tables S5, S7, S8

Table S4. Hyperparameters of the classification layer optimized for SSTDP (without weight norm.) on CSNN-128.

Hyperparameter	MNIST	Fashion-MNIST	CIFAR-10
V_{th}	150	150	400
g	0.07	0.05	0.0005
A^+	0.05	0.025	0.075
A^-	-0.0	-0.001	-0.01

Table S5. Hyperparameters of the classification layer optimized for SSTDP (with weight norm.) on CSNN-128.

Hyperparameter	MNIST	Fashion-MNIST	CIFAR-10
V_{th}	200	350	200
w_{norm}	0.2	0.2	0.2
g	0.06	0.03	0.0005
A^+	0.01	0.001	0.075
A^-	-0.05	-0.2	-0.075

Table S6. Hyperparameters of the classification layer optimized for S2-STDP (without weight norm.) on CSNN-128.

Hyperparameter	MNIST	Fashion-MNIST	CIFAR-10
V_{th}	300	350	550
g	0.0075	0.025	0.0025
A^+	0.05	0.1	0.1
A^-	-0.005	-0.005	-0.01

Table S7. Hyperparameters of the classification layer optimized for S2-STDP (with weight norm.) on CSNN-128.

Hyperparameter	MNIST	Fashion-MNIST	CIFAR-10
V_{th}	250	300	500
w_{norm}	0.3	0.3	0.3
g	0.03	0.02	0.005
A^+	0.005	0.005	0.001
A^-	-0.1	-0.075	-0.075

Table S8. Hyperparameters of the classification layer optimized for S2-STDP+PCN (with weight norm.) on CSNN-128.

Hyperparameter	MNIST	Fashion-MNIST	CIFAR-10
V_{th}	250	350	450
w_{norm}	0.3	0.3	0.3
g	0.02	0.005	0.001
A^{+}	0.001	0.0075	0.05
A^{-}	-0.1	-0.2	-0.2

3 ADDITIONAL EXPERIMENTS

3.1 S2-STDP addresses the issues of SSTDP

In the paper, we demonstrate on the MNIST dataset that S2-STDP successfully addresses the issues of SSTDP regarding the limited number of STDP updates per epoch and the saturation of firing timestamps toward the maximum firing time. In this section, we provide an additional experiment with similar results on the Fashion-MNIST dataset. Figure S1 shows that, at epoch 30, S2-STDP increases the update ratio from 16% to nearly 100%, reduces the average firing time from 0.99 to 0.89, and augments its standard deviation from 0.008 to 0.05. As a result, our proposed methods based on S2-STDP enable training convergence at higher accuracies. It is important to mention that the resolution of these issues is not attributed to the additional weight normalization mechanism employed by our methods. In Figure S2, we show on MNIST that SSTDP and S2-STDP exhibit similar behaviors with and without weight normalization, regarding update ratio, average firing time, and training accuracy.

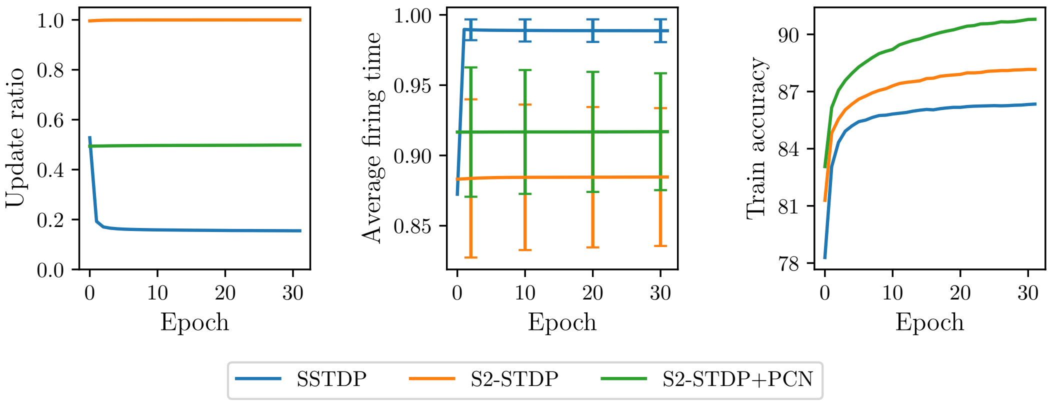

Figure S1: Update ratio, average firing time, and train accuracy per epoch in the classification layer trained on Fashion-MNIST. Our methods using S2-STDP significantly increase the number of updates per epoch and reduce the saturation of firing timestamps toward the maximum firing time. As a result, they enable training convergence at higher accuracies compared to SSTDP.

S2-STDP pushes neurons to fire closer to the average firing time, and hence, to each other, compared to SSTDP. While this behavior may seem undesirable to improve class separability, we show in the main paper that ensuring accurate control over the output firing timestamps is more important than maximizing the firing time difference between the target and non-target neurons. Figure S3 provides, for each class of

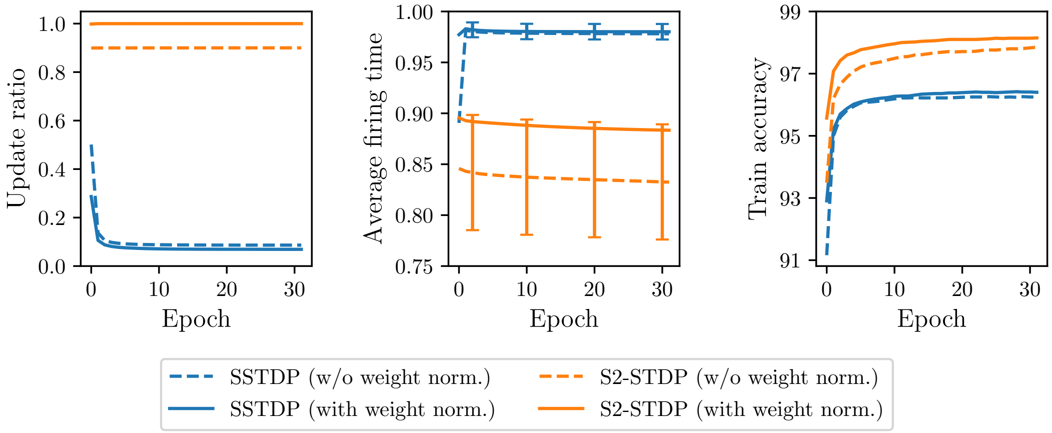

Figure S2: Update ratio, average firing time, and train accuracy per epoch in the classification layer trained on MNIST. S2-STDP successfully addresses the issues of SSTDP, with and without weight normalization.

the MNIST test set, the distribution of firing time differences between the first non-target neuron to fire and the target neuron, in the classification layer trained with SSTDP and S2-STDP. These distributions show that the firing time differences of S2-STDP tend to be significantly smaller compared to SSTDP but result in fewer misclassified samples.

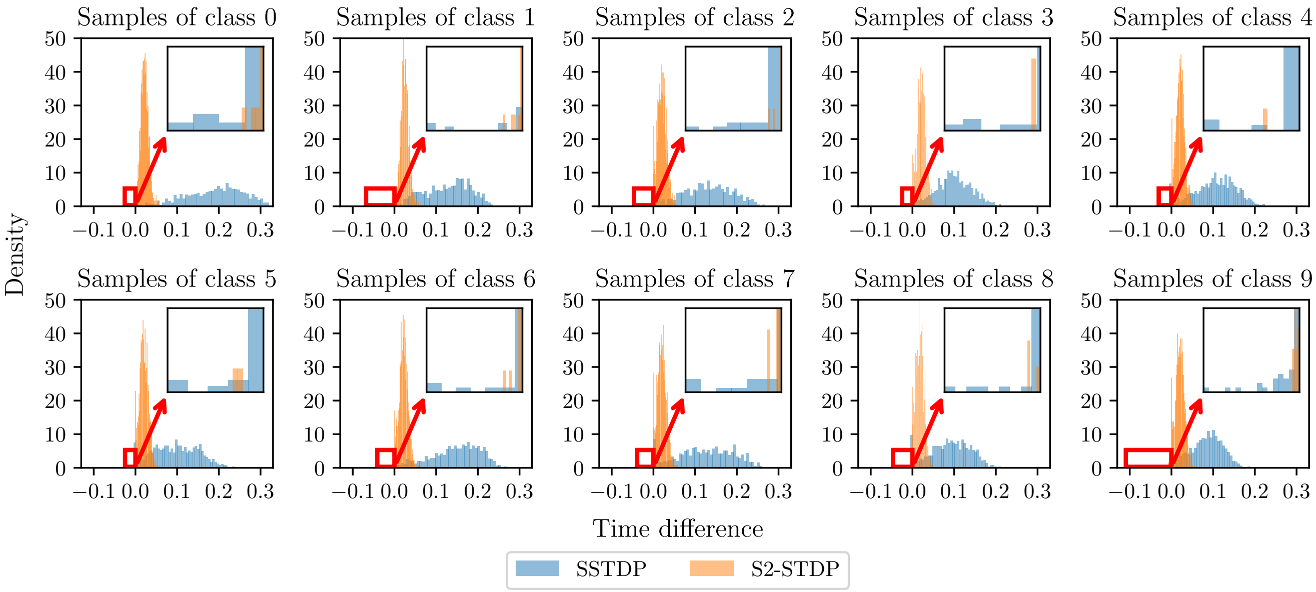

Figure S3: Distribution of firing time differences between the first non-target neuron to fire and the target neuron, in the classification layer on MNIST test samples. A negative time difference indicates that the target neuron fired after a non-target neuron, leading to misclassification of the sample. S2-STDP achieves a tighter distribution of time differences but results in fewer misclassified samples compared to SSTDP.

3.2 PCN enables neuron specialization

This work introduces the PCN architecture to improve the performance of our classification layer trained with S2-STDP. PCN represents each class by paired neurons and promotes specialization, such as one neuron learns to fire at the target desired timestamp and the other learns to fire at the non-target desired timestamp. The difference between the target and non-target firing timestamps is measured by the time gap g. Here, we analyze the actual firing time difference between paired neurons after training on the MNIST dataset. Figure S4 shows, for each class, the distribution of absolute firing time differences on test samples. As evidenced by the Gaussian shape distributions, paired neurons fire, on average, with a temporal interval of g. Hence, it provides evidence that PCN successfully enables neurons to specialize toward one of the two desired timestamps.

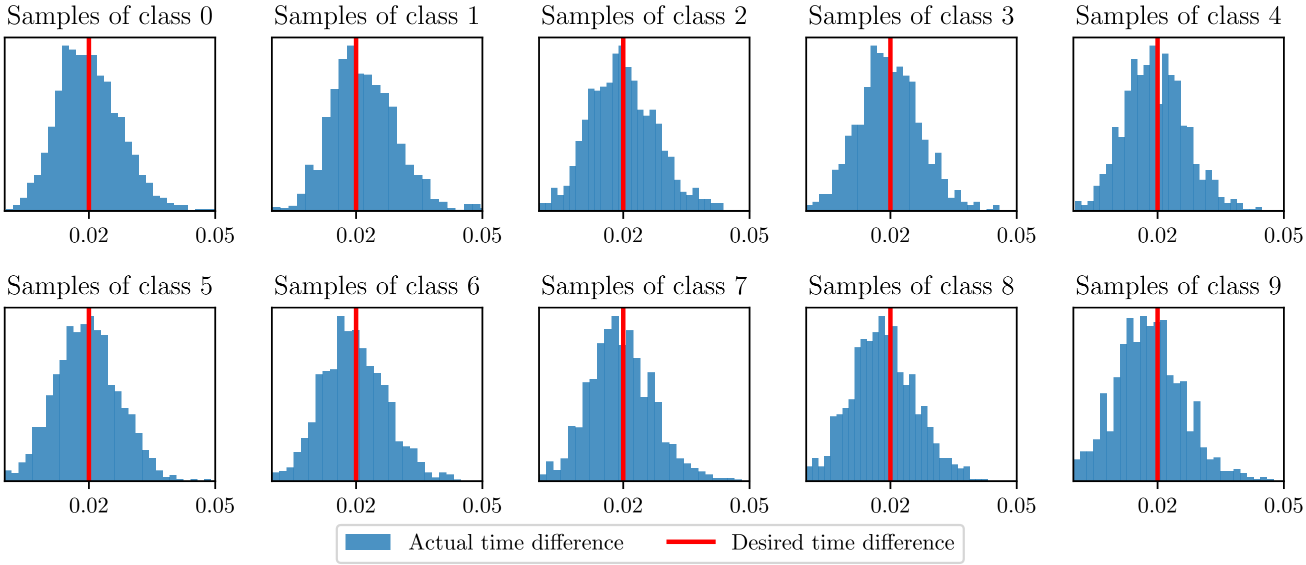

Figure S4: Distribution of absolute firing time differences between paired neurons on MNIST test samples, in the classification layer trained with S2-STDP+PCN.

In the paper, we provide evidence that neurons trained with S2-STDP in a PCN architecture are better at reaching the desired timestamps, especially for non-target samples. In this experiment, we show additional results on MNIST test samples to demonstrate this behavior. Figure S5 illustrates the distribution of time differences between neuron firing and desired timestamps, in the classification layer trained with S2-STDP and S2-STDP+PCN. As observed, S2-STDP+PCN achieves a significantly narrower distribution for non-target neurons, indicating that they fire, on average, closer to their desired timestamps. The distribution for target neurons is also slightly tightened, although to a lower extent. Nonetheless, the proportion of higher positive time differences (around 0.02), corresponding to target neurons firing after their desired timestamp, is reduced. It suggests that target neurons tend to fire farther away from non-target neurons, which may improve class separability.

3.3 Effect of weight normalization on accuracy

Training with S2-STDP involves weight normalization to ensure that neurons maintain a similar weight average during training, and hence, equal chances of activation. In this section, we evaluate the effect

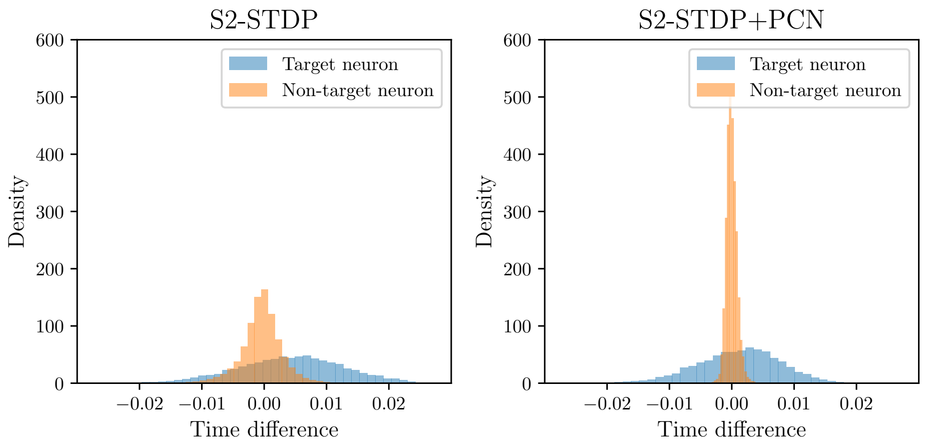

Figure S5: Distribution of time differences between neuron firing and desired timestamps, in the classification layer on MNIST test samples. Integrating a PCN architecture significantly tightens the non-target distribution, which indicates that neurons specializing toward non-target samples fire closer to their desired timestamps.

of weight normalization on accuracy. Figure S6 compares the accuracy of SSTDP and S2-STDP, with and without weight normalization. The hyperparameters have been optimized independently for each classification layer model. Also, note that the original SSTDP training method does not employ weight normalization. We observe that weight normalization consistently improves the accuracy of S2-STDP across all datasets. Conversely, with SSTDP, weight normalization is beneficial only on MNIST and CIFAR-10. Without normalization, S2-STDP and SSTDP achieve comparable performance on Fashion-MNIST and CIFAR-10. However, our proposed training method comprising S2-STDP and weight normalization consistently outperforms SSTDP, both with and without weight normalization. More importantly, S2-STDP leverages compatibility with the PCN architecture, which further improves the accuracy of S2-STDP across all datasets, and without requiring any additional hyperparameters.

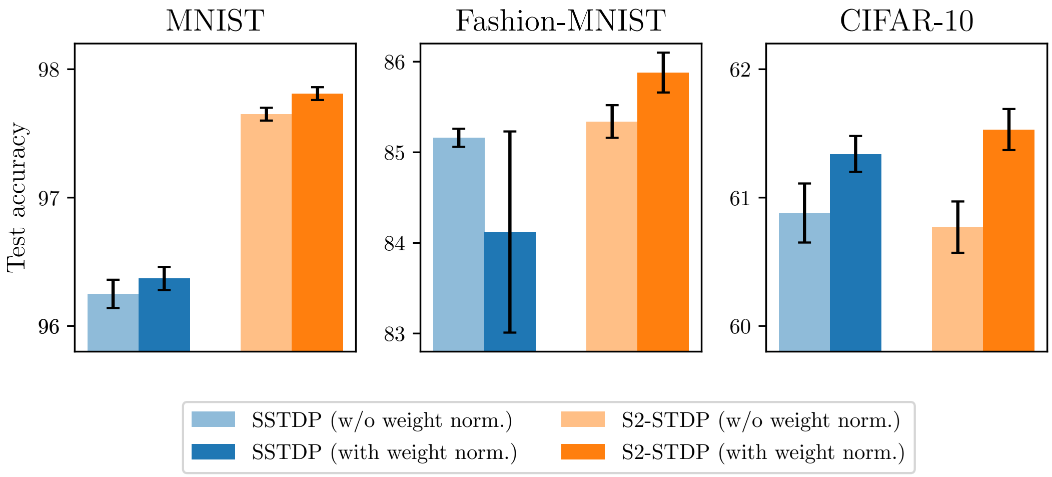

Figure S6: Accuracy of SSTDP and S2-STDP, both with and without weight normalization. Weight normalization consistently improves S2-STDP accuracy. As a result, our proposed training method comprising S2-STDP and weight normalization achieves higher accuracies compared to SSTDP.

3.4 Robustness against the hyperparameter set

In the main paper, we show on the Fashion-MNIST dataset that PCN can effectively improve the accuracy of S2-STDP irrespective of the hyperparameters used. Figures S7 and S8 provide additional evidence on the MNIST and CIFAR-10 datasets, respectively.

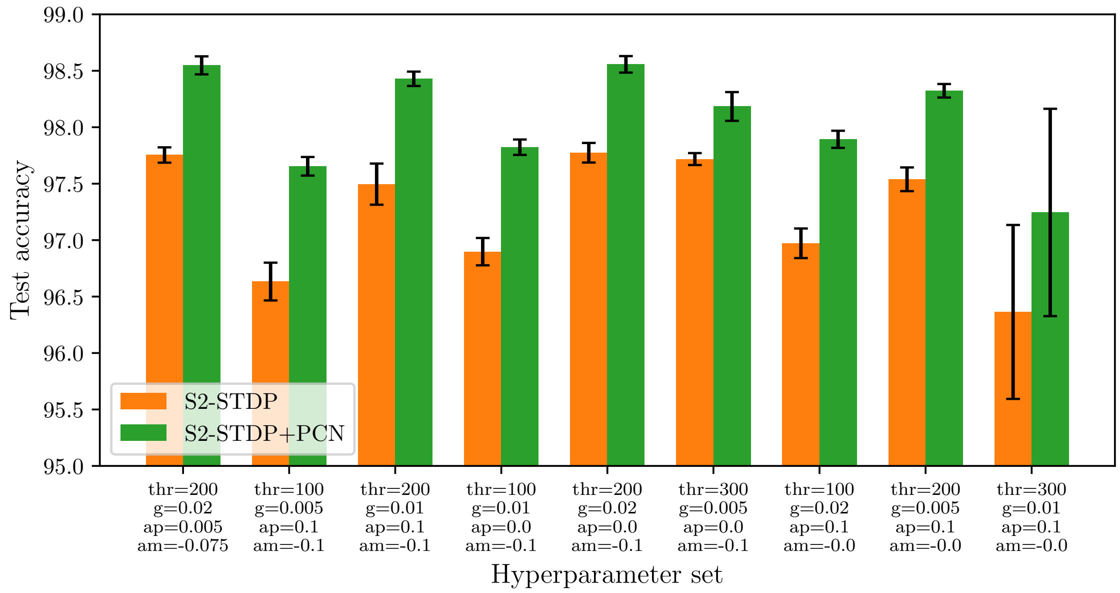

Figure S7: Accuracy of S2-STDP, with and without PCN, across different hyperparameter sets on MNIST. PCN always improves S2-STDP performance, without introducing any additional hyperparameters.

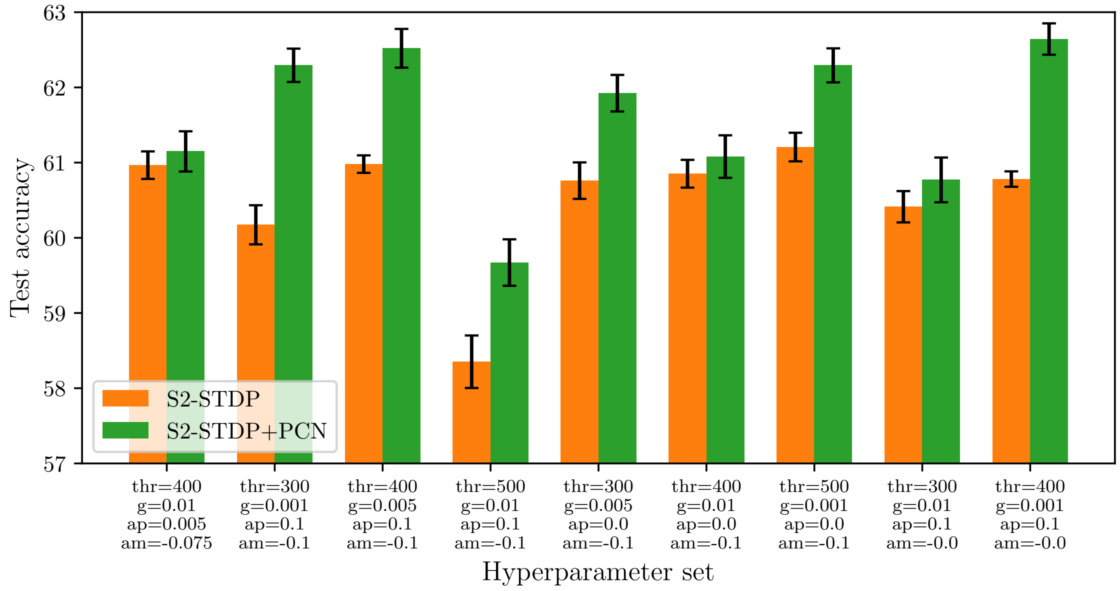

Figure S8: Accuracy of S2-STDP, with and without PCN, across different hyperparameter sets on CIFAR-10. PCN always improves S2-STDP performance, without introducing any additional hyperparameters.

REFERENCES

Falez, P., Tirilly, P., Marius Bilasco, I., Devienne, P., and Boulet, P. (2019). Multi-layered Spiking Neural Network with Target Timestamp Threshold Adaptation and STDP. In *International Joint Conference on Neural Networks*
